# The Clinicopathological Risk Factors in Renal Cell Cancer for the Oncological Outcomes Following Nephron-Sparing Surgery: A PRISMA Systematic Review and Meta-Analysis

**DOI:** 10.3389/fonc.2020.00286

**Published:** 2020-03-06

**Authors:** Lijin Zhang, Bin Wu, Zhenlei Zha, Wei Qu, Hu Zhao, Jun Yuan

**Affiliations:** ^1^Department of Urology, Affiliated Jiang-yin Hospital of the Southeast University Medical College, Jiangyin, China; ^2^Department of Pharmacy, Affiliated Jiang-yin Hospital of the Southeast University Medical College, Jiangyin, China

**Keywords:** renal cell cancer, nephron-sparing surgery, clinicopathological, oncological outcome, meta-analysis

## Abstract

**Background and Objectives:** Published data from individual studies present conflicting evidence about the relationship between clinicopathological risk factors and oncological outcomes in renal cell cancer (RCC) following nephron-sparing surgery (NSS). This study was conducted to explore the potential risk factors for RCC progress after NSS.

**Methods:** Studies published in PubMed, Web of Science, and EMBASE were systematically reviewed from inception to March 2019 to determine risk factors for RCC following NSS. The predictive ability of identified predictors was assessed by hazard ratios (HRs) with 95% confidence intervals (CIs). A fixed-effect or random-effect was used to pool the estimates. Subgroup analyses were performed to explore the source of heterogeneity.

**Results:** Seventeen studies including 38,522 patients with RCC were analyzed. The meta-analysis indicated that positive surgical margin (pooled HR = 1.47; 95% CI:1.24–1.73; *P* < 0.001), higher Fuhrman grade (pooled HR = 1.58; 95% CI:1.10–2.28; *P* = 0.013), higher pathological stage (pooled HR = 1.72; 95% CI:1.40–2.12; *P* < 0.001) and large tumor size (pooled HR = 1.09; 95% CI:1.03–1.16; *P* = 0.003) were significantly associated with recurrence risk. However, age (pooled HR = 1.00; 95% CI: 1.00–1.01; *P* = 0.257), sex (male vs. female) (pooled HR = 1.04; 95% CI: 0.89–1.21; *P* = 0.605) and surgical approach (laparoscope vs. open) (pooled HR = 0.80; 95% CI: 0.59–1.07; *P* = 0.129) had no effect on recurrence after NSS. In addition, we found that positive surgical margin was significantly associated with recurrence-free survival (pooled HR = 1.87; 95% CI: 1.32–2.66; *P* < 0.001) and overall mortality (pooled HR = 1.15; 95% CI: 1.07–1.23; *P* < 0.001), as well as large tumor size for recurrence-free survival (pooled HR = 1.18; 95% CI: 1.06–1.30; *P* = 0.002)and overall mortality (pooled HR = 1.01; 95% CI: 1.00–1.02; *P* = 0.004).

**Conclusions:** Unfavorable pathological characteristics were distinctly related to worse oncological outcomes in RCC patients following NSS. These results may contribute to proposed prediction models for RCC patients to aid in counseling and risk stratification.

## Background

Renal cell cancer (RCC) is the third most fatal genitourinary malignancy, accounting for 2–3% of all adult malignancies in humans (1). With the widespread use of cross-sectional imaging in the last decades, more renal tumors are expected to be detected as local lesions. Although the conventional treatment for RCC is radical nephrectomy (RN), patients with RN have substantial risk of progression to future renal insufficiency (2). Hence, nephron-sparing surgery (NSS), involving preservation of normal kidney parenchyma, has become widely accepted as the standard treatment of small renal masses (3).

Traditionally, nephron-sparing surgery includes partial nephrectomy and simple enucleation. Current evidence demonstrates that NSS leads to equal oncological outcomes compared to RN for pathologically staged T1- T2 tumors (4, 5). However, 20–40% of patients experience local recurrence or distant metastasis after NSS (6, 7). This has led to attempts to identify clinicopathological factors to assist clinical decision-making and patient counseling. Nowadays, some prognostic parameters, such as TNM staging system (8), tumor size (9), histological subtype (10) and Fuhrman grade (11)have been developed to predict disease recurrence or survival outcomes. However, the prognostic value of these parameters are controversial according to published studies, and there is no consensus about which patients are at greater risk to develop recurrence or distant metastasis.

These controversies could be a result of differences in limited sample sizes and (or) individual variations. To date, there is no comprehensive study containing the clinicopathological variables for RCC. In this setting, we searched the relevant studies and conducted this meta-analysis in order to assess the potential prognostic factors for oncologic outcomes of recurrence, recurrence-free survival (RFS) and overall mortality (OM) after NSS in RCC patients.

## Materials and Methods

### Search Strategy

Following the Preferred Reporting Items for Systematic Reviews and Meta-analyses (PRISMA) protocols (12), relevant studies were searched through the electronic databases of PubMed, EMBAS, and Web of Science up to March, 2019.The following MeSH terms and text words were used in combination: “renal cell cancer,” “nephron-sparing surgery,” “partial nephrectomy,” “simple enucleation,” “prognosis,” “clinicopathological,” and “survival.” We also searched relevant studies and reviews by manually screening the references list. The search was restricted to studies of human subjects written in English. Institutional Review Board approval was not required for this study.

### Inclusion and Exclusion Criteria

The criteria for inclusion were listed as follows: (1) patients with a diagnosis of RCC was pathologically confirmed; (2) the study considered NSS as a primary treatment; (3) the study included clinicopathological factors for oncological outcomes in RCC; and (4) the authors offered the hazard ratios (HRs) and their 95% confidence intervals (CIs) in the paper. The exclusion criteria for the primary studies included following criteria: (1) duplicate publications; (2) studies which did not provide sufficient data to acquire HRs and 95%CIs; (3) non-original articles (e.g., reviews, letters, case reports, and author's reply). When multiple articles were published about the same population, the article reporting the most complete data would be used.

### Data Extraction and Quality Assessment

Two authors (ZlZ and HZ) independently extracted data from each included paper. Any disagreement was resolved by discussing with the senior author (BW). The following extracted information was collected and recorded in standardized form: first author, year of publication, ethnicity, number of patients, recruitment period, age, sex, type of NSS, histopathological information, follow-up time, and HRs for survival outcome (recurrence, RFS, and OM) with its 95% CIs. If one study reported results using both multivariate and univariate analysis, we choose the results from multivariate analysis, as it accounts for confounding factors and is more precise.

The Newcastle–Ottawa Quality Assessment Scale (NOS) (13) was applied to assess the methodological quality of each included study. Selection of cohorts, comparability, and ascertainment of outcomes were involved in this assessment scale. Only studies with an NOS score >6 were defined as high quality and finally included.

### Statistical Analysis

The software Stata version 12.0 (STATA Corporation, College Station, TX) was used to perform the meta-analysis of the included articles. HRs with their 95% CIs extracted from each publication were applied to calculate pooled HRs. Pooled HRs were used to identify the correlation between clinicopathological risk factors and patient survival. A pooled HR >1 indicated a poorer survival for the patients with a certain clinicopathological feature. The *Chi*^2^ and *I*^2^ statistic were performed to evaluate inter-study heterogeneity. The value of *P*_heterogeneity_ > 0.1 and I^2^ < 50% represents low-level heterogeneity. If *P*_heterogeneity_ > 0.1 and *I*^2^ < 50%, a fixed-effects model was applied; otherwise, a random-effects model was applied.

Subgroup and meta-regression analysis based on geographical region, publication year, sample size, and follow-up duration were performed for recurrence analysis to identify the source of heterogeneity. Funnel plots and Begg's test were applied to assess potential publication bias. Sensitivity analysis was also conducted by deleting one single study to measure the reliability of the pooled results. Two-sided value of *p* < 0.05 was considered statistically significant.

## Results

### Search Results

**Figure 1:**
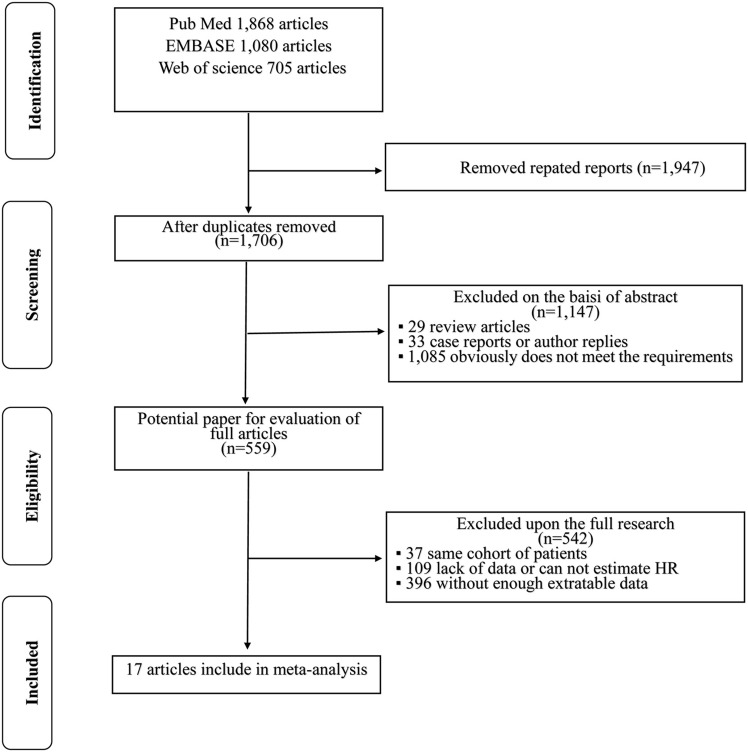
Flow chart of the studies selection process in the meta-analysis.

The process of searching articles is presented as a flow diagram in Figure 1. A total of 3,653 potentially relevant studies were identified through the primary study searching. Of all identified records, 1,947 were excluded due to duplicate studies. After title and/or abstracts scanning, 559 articles remained for full-text assessment. Then 543 articles were further excluded by the inclusion criteria. Ultimately, 17 published retrospective studies that met our inclusion criteria were included in this meta-analysis (14–30), containing a total of 38,522 patients (ranging from 69 to 20,762).

### Characteristics of the Studies

The main clinicopathological characteristic of included studies are presented in Table 1 and Table 2. Totally, all the studies were written in English and published from 2002 to 2018. The median follow-up intervals were from 19 to 102 months. In these studies, nine studies were reported from American countries (USA, Canada, Mexico, Argentina), four from Asian countries (Korea, Japan, Indian), three from European countries (Italy, France), and one being a multi-center study. Thirteen studies reported the correlation between recurrence and clinicopathological features; the remaining three studies reported RFS and/or OM and clinicopathological details. The results of the NOS assessment are summarized in Supplementary Table S1; the quality scores of the studies varied from 6 to 8, with a mean of 7.5.

**Table 1:** The main characteristics of the eligible studies.

**References**	**Country**	**Recruitment period**	**No. of patients**	**Age (years)**	**Gender (m/f)**	**Follow-up (months)**	**Surgical operation (open vs. minimally invasive)**
Wood et al. (14)	USA	2000–2014	207	NA	111/86	Median (range) 23 (2–107)	PN(179 vs. 28)
Tellini et al. (15)	Italy	1983–2014	459	Mean ± SD 60.7 ± 12.7	328/131	Median (IQR) 96 (74–131)	NSS
Shum et al. (16)	Indian	2004–2009	20,762	NA	12,745/8,017	Median 70.3	PN
Marchinena et al. (17)	Argentina	2010–2015	314	Mean ± SD 58.3 ± 12	218/96	Median (IQR) 24 (12–40)	NSS(142 vs. 172)
Yoo et al. (18)	Korea	1998–2012	843	Mean ± SD 53.3 ± 11.7	636/207	Median 67	PN(468 vs. 375)
Bansal et al. (19)	Canada	2011–2014	1,103	Median (range) 61 (53–70)	654/389	Median (IQR) 19 (5–42)	PN(599 vs. 504)
Shah et al. (20)	Muti-centers	2006–2013	1,240	Mean ± SD 59.1 ± 11.9	832/408	Median (IQR) 33 (15–57)	PN
Nguyen et al. (21)	USA	2006–2014	1,668	Median (IQR) 60 (51–68)	1,131/555	Median (IQR) 33.6 (13.2–60)	PN
Maurice et al. (22)	USA	2003–2006	6,038	Median (IQR) 58 (49–67)	3,644/2,394	Median (IQR) 71 (56–85)	PN
Lee et al. (23)	Korea	1997–2014	1,367	NA	1,016/351	Median (IQR) 54 (29–81)	PN
Zargar-Shoshtari et al. (24)	Mexico	1999–2012	505	Median (range) 61 (22–88)	314/191	Median (IQR) 38.3 (6–88)	NSS(377 vs. 128)
Minervini et al. (25)	Italy	2005–2011	304	Mean ± SD 63 ± 13	192/112	Median (range) 52 (12–96)	SE
Bigot et al. (26)	France	1998–2012	168	Median (range) 59 (20–85)	102/66	Mean (range) 30 (1–254)	NSS(153 vs.)
Lane et al. (27)	USA	1999–2008	1,616	Median (IQR) 61 (52–70)	1,021/595	Median (IQR) 58.8 (39.6–81.6)	PN(626 vs. 395)
Yossepowitch et al. (28)	USA	1972–2005	1,390	Median (IQR) 61 (52–69)	954/436	Median (IQR) 40.8 (16.8–70.8)	PN
Senga et al. (29)	Japan	1990–1999	469	NA	361/108	Mean (range) 48.2 (1–158)	PN
Castilia et al. (30)	USA	1976–1988	69	Mean (range) 61 (36–85)	47/22	Median 102	NSS

*m/f, male/female; SD, standard deviation; IQR, inter quartile range; NA, data not applicable; PN, partial nephrectomy; SE, simple enucleation; NSS, nephron-sparing surgery*.

**Table 2:** The main pathologic features of the eligible studies.

**Study**	**Staging system**	**Grading system**	**PSM / NSM**	**Stage1-2/ 3-4**	**Grade 1-2/ 3-4**	**Tumor size (cm)**
Wood et al. (14)	2010 AJCC	Furman	12/195	193/14	125/82	NA
Tellini et al. (15)	2010 AJCC	Furman	27/432	441/18	NA	Mean ± SD3.1 ± 1.3
Shum et al. (16)	2010 AJCC	Furman	1,278/19,484	NA	14,495/3,887	NA
Marchinena et al. (17)	2002 AJCC	Furman	22/292	314/10	258/7	Median (IQR)2.9 (2.1–3.8)
Yoo et al. (18)	NA	Furman	NA	843/0	583/260	Mean ± SD2.2 ± 0.8
Bansal et al. (19)	NA	Furman	71/972	1,036/67	829/274	Median3.0
Shah et al. (20)	2002 AJCC	Furman	97/1,143	1,240/0	927/313	Mean ± SD3.2 ± 1.7
Nguyen et al. (21)	2009 AJCC	NA	NA	1,450/218	NA	Median (IQR)3.0 (2.0–4.0)
Maurice et al. (22)	2010 AJCC	Furman	302/6,038	5,898/140	5,133/905	Median (IQR)2.5 (1.9–3.5)
Lee et al. (23)	2009 AJCC	Furman	10/1,357	1,324/43	927/440	NA
Zargar-Shoshtari et al. (24)	2009 AJCC	Furman	3/502	488/17	258/247	Median (range)3 (0.2–13)
Minervini et al. (25)	2009 AJCC	Furman	NA	279/25	276/28	Mean (range)3.4 (1–12.5)
Bigot et al. (26)	2009 AJCC	Fuhrman	14/154	107/19	63/163	Median (range)8 (7–18)
Lane et al. (27)	NA	Furman	7/1609	NA	1,200/416	Median (IQR)3.0 (2.2-4.0)
Yossepowitch et al. (28)	NA	NA	77/1,313	1,311/79	NA	Median (IQR)3.0 (2.2-4.3)
Senga et al. (29)	1997 AJCC	Furman	NA	NA	462/7	NA
Castilia et al. (30)	1997 AJCC	Furman	NA	46/23	61/8	Median (range)4.4 (1-11.3)

*SD, standard deviation; IQR, inter quartile range; NA, data not applicable*.

### Survival Outcome

**Figure 2:**
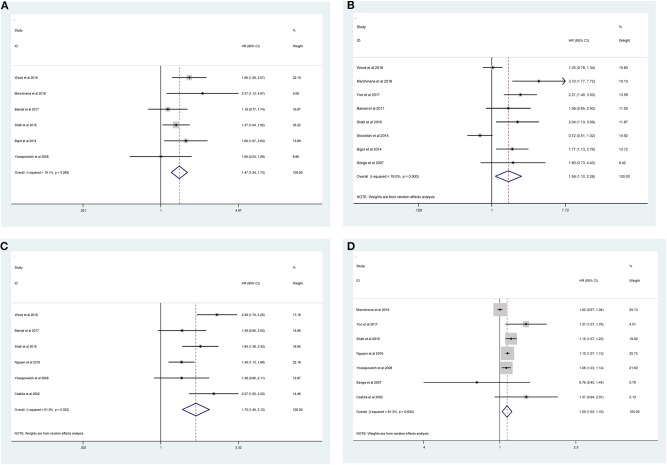
Forest plot of hazard ratio for the association between clinicopathological features and recurrence risk in RCC patients following NSS: **(A)** positive surgical margin; **(B)** higher Fuhrman grade; **(C)** higher pathological stage; **(D)** large tumor size.

**Figure 3:**
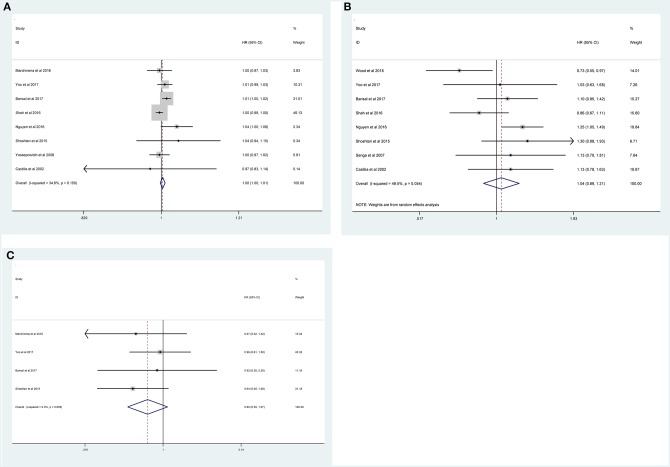
Forest plot depiction of the association between clinicopathological factors and recurrence risk in RCC patients following NSS: **(A)** age; **(B)** sex; **(C)** surgical approach.

Fourteen studies with 10,106 patients was enrolled to disclose the clinicopathological factors in patients with NSS.As shown in Figure 2, positive surgical margin(PSM) (pooled HR = 1.47; 95% CI:1.24–1.73; *P* < 0.001, Figure 2A), higher Fuhrman grade (pooled HR = 1.58, 95% CI:1.10–2.28, *P* = 0.013, Figure 2B), higher pathological stage (pooled HR = 1.72; 95% CI:1.40–2.12; *P* < 0.001, Figure 2C) and large tumor size (pooled HR = 1.09; 95% CI:1.03–1.16; *P* = 0.003, Figure 2D) were significantly associated with higher recurrence risk. However, age (pooled HR = 1.00; 95% CI: 1.00–1.01; *P* = 0.257, Figure 3A), sex (male vs. female) (pooled HR = 1.04, 95% CI: 0.89–1.21, *P* = 0.605, Figure 3B) and surgical approach (laparoscope vs. open) (pooled HR = 0.80, 95% CI: 0.59–1.07, *P* = 0.129, Figure 3C) had no effect on recurrence.

We also investigated the potential clinicopathological risk factors for RFS and OM in three studies with 28,416 patients. As shown in Table 3, PSM (pooled HR = 1.87,95% CI:1.32–2.66, *P* < 0.001, Supplementary Figure S1a), higher Fuhrman grade (pooled HR = 1.75, 95% CI:1.30–2.37, *P* < 0.001, Supplementary Figure S1b), higher pathological stage (pooled HR = 2.21, 95% CI:1.64–2.97, *P* < 0.001, Supplementary Figure S1c) and large tumor size (pooled HR = 1.18, 95% CI:1.06–1.30, *P* = 0.002, Supplementary Figure S1d) were significantly associated with poor RFS. However, age (pooled HR = 1.01, 95% CI: 0.98–1.04, *P* = 0.488, Supplementary Figure S1e) and sex (male vs. female) (pooled HR = 1.04, 95% CI: 0.90–1.21, *P* = 0.592, Supplementary Figure S1f) had no relationship with RFS. Also, PSM (pooled HR = 1.15, 95% CI: 1.07–1.23, *P* < 0.001, Supplementary Figure S2a) and large tumor size (pooled HR = 1.01, 95% CI: 1.00–1.02, *P* = 0.004, Supplementary Figure S2b) indicated a strong association with OM. Meanwhile, OM has no association with sex (male vs. female) (pooled HR = 1.08, 95% CI: 0.96–1.23, *P* = 0.193, Supplementary Figure S2c). Taken together, the results demonstrated that worse pathological features may be considered as significant biomarkers for prognosis of patients following NSS.

**Table 3:** The pooled HR and 95% CIs for the prognostic factors in RFS and OM.

**Analysis specification**	**No. of studies**	**Study heterogeneity**		**Effects model**	**Pooled HR(95% CI)**	***P*-Value**
		***I*^**2**^ (%)**	***P*_**heterogeneity**_**			
**RFS**						
Age	2	79.3	0.028	Random	1.01(0.98, 1.04)	0.488
Tumor size	3	61	0.077	Random	1.18(1.06, 1.30)	0.002
Sex (male vs. female)	2	0	0.587	Fixed	1.04(0.90, 1.21)	0.592
Fuhrman grade	3	0	0.524	Fixed	1.75(1.30, 2.37)	<0.001
Pathological stage	3	0	0.423	Fixed	2.21(1.64, 2.97)	<0.001
PSM	2	0	0.355	Fixed	1.87(1.32, 2.66)	<0.001
**OM**						
Sex (male vs. female)	2	55.2	0.315	Random	1.08(0.96, 1.23)	0.193
Tumor size	2	0	0.347	Fixed	1.01(1.00, 1.02)	0.004
PSM	2	0	0.860	Fixed	1.15(1.07, 1.23)	<0.001

### Subgroup Analysis

Because the number of studies that evaluated RFS and OM was relatively small, we only conducted subgroup analysis for recurrence. The results of this subgroup analysis again suggested that PSM, bigger tumor size, higher Fuhrman grade and pathological stage were prognostic factors for RCC, despite certain heterogeneity among some groups (Table 4). Notably, heterogeneity for recurrence was significantly decreased in some models, such as geographical region in Asia, number of patients ≥800 cases, and year of publication before 2016.

**Table 4:** Summary and subgroup analysis for the prognostic factors in recurrence risk.

**Analysis specification**	**No. of studies**	**Study heterogeneity**		**Effects model**	**Pooled HR(95% CI)**	***P*-value**
		***I*^**2**^ (%)**	***P*_***heterogeneity***_**			
**Age**						
Overall	8	34.8	0.150	Fixed	1.00(1.00, 1.01)	0.257
**Surgical approach (laparoscope vs. open)**						
Overall	4	0	0.658	Fixed	0.80(0.59, 1.07)	0.129
**PSM**						
Overall	6	19.1	0.289	Fixed	1.47(1.24, 1.73)	<0.001
**Sex (Male vs. Female)**						
Overall	8	49.5	0.054	Random	1.04(0.89, 1.21)	0.605
**Geographical region**						
Asia	2	59.1	0.118	Random	0.87(0.57, 1.33)	0.514
Non-Asian	6	20.2	0.281	Fixed	1.11(0.97, 1.26)	0.128
**Year of publication**						
≥2016	5	67.7	0.015	Random	0.98(0.80, 1.22)	0.887
<2016	3	0	0	Fixed	1.19(0.94, 1.50)	0.151
**No. of patients**						
≥800	4	46.5	0.133	Random	1.07(0.89, 1.28)	0.461
<800	4	57.8	0.068	Random	1.02(0.77, 1.36)	0.886
**Median follow-Up**						
≥40 months	3	0	0.953	Fixed	1.10(0.86, 1.41)	0.443
<40 months	5	70.7	0.009	Random	1.02(0.82, 1.27)	0.858
**Fuhrman Grade**						
Overall	8	78	<0.001	Random	1.58(1.10, 2.28)	0.013
**Geographical region**						
Asia	2	28	0.239	Fixed	1.15(0.74, 1.79)	0.541
non-Asian	6	81.4	<0.001	Random	1.73(1.07, 2.80)	0.025
**Year of publication**						
≥2016	5	76.7	0.002	Random	1.84(1.16, 2.91)	0.1
<2016	3	81.9	0.004	Random	1.26(0.63, 2.52)	0.523
**No. of patients**						
≥800	3	0	0.689	Fixed	1.98(1.45, 2.71)	<0.001
<800	5	81.5	<0.001	Random	1.42(0.88, 2.29)	0.155
**Median follow-Up**						
≥40 months	2	0	0.692	Fixed	2.12(1.41, 3.19)	<0.001
<40 months	6	80.1	<0.001	Random	1.48(0.97, 2.24)	0.067
**Pathological stage**						
Overall	6	61.9	0.022	Random	1.72(1.40, 2.12)	<0.001
**Geographical region**						
Asia	1					
Non-Asian	5	44.7	0.124	Random	1.60(1.32, 1.93)	<0.001
**Year of publication**						
≥2016	4	69.4	0.020	Random	1.69(1.31, 2.19)	<0.001
<2016	2	65.4	0.089	Random	1.79(1.10, 2.90)	0.019
**No. of patients**						
≥800	4	2.5	0.380	Fixed	1.49(1.29, 1.72)	<0.001
<800	2	0	0.845	Fixed	2.33(1.83, 2.97)	<0.001
**Median follow-Up**						
≥40 months	2	65.4	0.089	Random	1.79(1.10, 2.90)	0.019
<40 months	4	69.4	0.020	Random	1.69(1.31, 2.19)	<0.001
**Tumor size**						
Overall	7	81.3	<0.001	Random	1.09(1.03, 1.16)	0.003
**Geographical region**						
Asia	1					
Non-Asian	6	83.9	<0.001	Random	1.10(1.03, 1.16)	0.002
**Year of publication**						
≥2016	4	89.7	<0.001	Random	1.10(1.02, 1.18)	0.015
<2016	3	26.0	0.259	Fixed	1.10(0.92, 1.31)	0.290
**No. of patients**						
≥800	4	38.3	0.182	Fixed	1.11(1.07, 1.15)	<0.001
<800	3	40.5	0.186	Fixed	1.05(0.84, 1.31)	0.677
**Median follow-Up**						
≥40 months	4	49.8	0.113	Fixed	1.17(0.98, 1.40)	0.091
<40 months	3	92.1	<0.001	Random	1.08(1.00, 1.16)	0.045

### Sensitivity Analysis and Publication Bias

**Figure 4:**
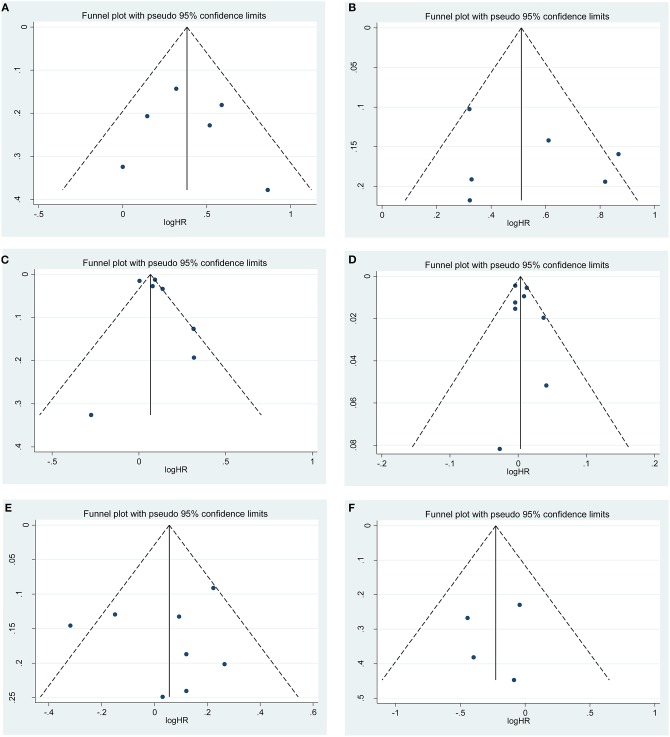
Funnel plots of publication bias on the correlation between clinicopathological features and recurrence risk in this meta-analysis: **(A)** positive surgical margin; **(B)** higher pathological stage; **(C)** large tumor size; **(D)** age; **(E)** sex; **(F)** surgical approach.

Sensitivity analysis was performed to examine the stability of the current meta-analysis. As shown in Supplementary Figure S3, the overall HRs for recurrence were found to be stable and not altered by removal of any single study. Funnel plots and Begg's test were used to assess the publication bias in this meta-analysis. Funnel plots for clinicopathological risk factors and recurrence are shown in Figure 4. Using Begg's test, no obvious publication bias was found regarding PSM (*p* = 0.807, Figure 4A), higher pathological stage (*p* = 0.483, Figure 4B), large tumor size (*p* = 0.543, Figure 4C), age (*p* = 0.524, Figure 4D), sex (*p* = 0.728, Figure 4E) and surgical approach (*p* = 0.781, Figure 4F). However, a slight publication bias existed in higher Fuhrman grade (*p* = 0.043). Because the number of included studies was limited, the publication bias for RFS and OM were not assessed.

## Discussion

RCC is one of the most common cancers, with a major worldwide clinical and public health burden. Improved diagnostics have resulted in the increasing discovery of low stage renal tumors. Although the application of standardized NSS treatment has significantly improved the prognosis of early-stage RCC (31), tumor recurrence and metastasis is still a serious challenge for doctors and patients. RCC have different clinical and biological characteristics, with an extremely heterogeneous oncological outcome (32). Therefore, it is necessary to find prognostic predictors to distinguish high-risk patients and improve the overall clinical outcome in RCC.

Several stratification nomograms have been developed to predict the progression and prognosis after NSS in the postoperative setting. Clinicopathological features that are associated with survival outcome have been intensively studied in the past few years. The Kattan nomogram (33), which contain both clinical and pathological parameters, was the first reported classification system to predict the probability of recurrence in RCC patients. Similarly, the University of California, Los Angeles, Integrated Staging System (UISS) (34) and TNM stage, tumor size, Fuhrman grade, and tumor necrosis (SSIGN) score (35) developed from the Mayo Clinic were conducted to predicting the oncological outcome.

However, most studies investigating the prognosis predictors for RCC following NSS are restricted by single-center design, small sample size, or ethnic differences. For example, the Kattan nomogram has low predictive accuracy in French (36) and Japanese patients (37). Therefore, the precision of the models may be unsatisfactory, and the proposed nomograms still need to be externally validated before clinical application. Moreover, some researchers have reported that other clinicopathological factors such as sex, age, and race may also influence the RCC patients' outcomes (38, 39). Neglecting these prognostic parameters may reduce the accuracy of survival predictions. Hence, we aimed to evaluate the significant clinicopathological variables of oncologic outcomes after NSS from a meta-analysis based on all the available data.

To the best of our knowledge, the present meta-analysis is the first comprehensive study to analyze the prognostic value of clinicopathological parameters in RCC patients after NSS. The pooled results indicated that large tumor size, high Fuhrman grade, higher pathological stage, and PSM at surgery were unfavorable predictors for both recurrence and RFS. Similarly, there was a significant correlation of PSM and large tumor size with worse OM in RCC patients. Generally, in the subgroup analysis for recurrence, these adverse features were still an important prognostic marker in RCC patients, regardless of race, publication year, sample size and follow-up duration. Since these clinicopathologic factors are easily obtained, they can be used to guide patient counseling and risk stratification after NSS.

Several limitations in this meta-analysis should be acknowledged. First, all included studies were retrospectively performed, and data extracted from those studies may have led to inherent bias. Although we extract the results from multivariate models, there still confounding factors can't be controlled. Second, significant heterogeneity was found among studies. However, subgroup analyses showed that the heterogeneity diminished in some groups. Moreover, the stability of our results was confirmed by sensitivity analysis. Third, only published articles were included, and they were all written in English. Therefore, potential selection bias may existed in our study. Fourth, the number of included studies of RFS and OM were smaller, and relevant data could not be obtained for further analysis. Additionally, publication bias was detected in higher Fuhrman grade for recurrence. It is well known that papers with positive results are more likely to be published.

## Conclusion

The present study demonstrated that PSM, large tumor size, higher pathological stage and Fuhrman grade were closely associated with poorer prognosis in patients following NSS. However, age, sex and surgical approach were not associated with recurrence, RFS and OM. The findings in our study may help clinicians to identify high-risk patients and formulate treatment decisions. Patients with those with risk factors should be subjected to closer surveillance. Due to the limitations in this meta-analysis, further well-designed studies are required to clarify our results.

## Data Availability Statement

All datasets generated for this study are included in the article/Supplementary Material.

## Author Contributions

LZ and BW: conceptualization. ZZ, WQ, and BW: literature search. JY and HZ: data analysis. LZ: writing—original draft. BW: writing—review and editing. All authors approved the final manuscript.

### Conflict of Interest

The authors declare that the research was conducted in the absence of any commercial or financial relationships that could be construed as a potential conflict of interest.

## Supplementary Material

Supplementary Figure S1 Meta-analysis for the pooled HRs of RFS: **(a)** positive surgical margin; **(b)** higher Fuhrman grade; **(c)** higher pathological stage; **(d)** large tumor size; **(e)** age; **(f)** sex.

Supplementary Figure S2 Meta-analysis for the pooled HRs of OM: **(a)** positive surgical margin; **(b)** large tumor size; **(c)** sex.

Supplementary Figure S3 Sensitivity analysis on the relationship between clinicopathological features and recurrence risk: **(a)** positive surgical margin; **(b)** higher Fuhrman grade; **(c)** higher pathological stage; **(d)** large tumor size; **(e)** age; **(f)** sex; **(g)** surgical approach.

## Abbreviations

RCC: renal cell cancer
NSS: nephron-sparing surgery
PRISMA: Preferred Reporting Items for Systematic Reviews and Meta-Analyses
NOS: Newcastle Ottawa scale
HRs: Hazard ratios
CIs: corresponding 95% confidence intervals
RFS: recurrence-free survival
OM: overall mortality
PSM: positive surgical margin
PN: partial nephrectomy
SE: simple enucleation.
