# Supplementary figures and images for: The Clinicopathological Risk Factors in Renal Cell Cancer for the Oncological Outcomes Following Nephron-Sparing Surgery: A PRISMA Systematic Review and Meta-Analysis
Source: Front Oncol. 2020 Mar 6;10:286. doi: 10.3389/fonc.2020.00286 (PMC7067827; doi:10.3389/fonc.2020.00286)

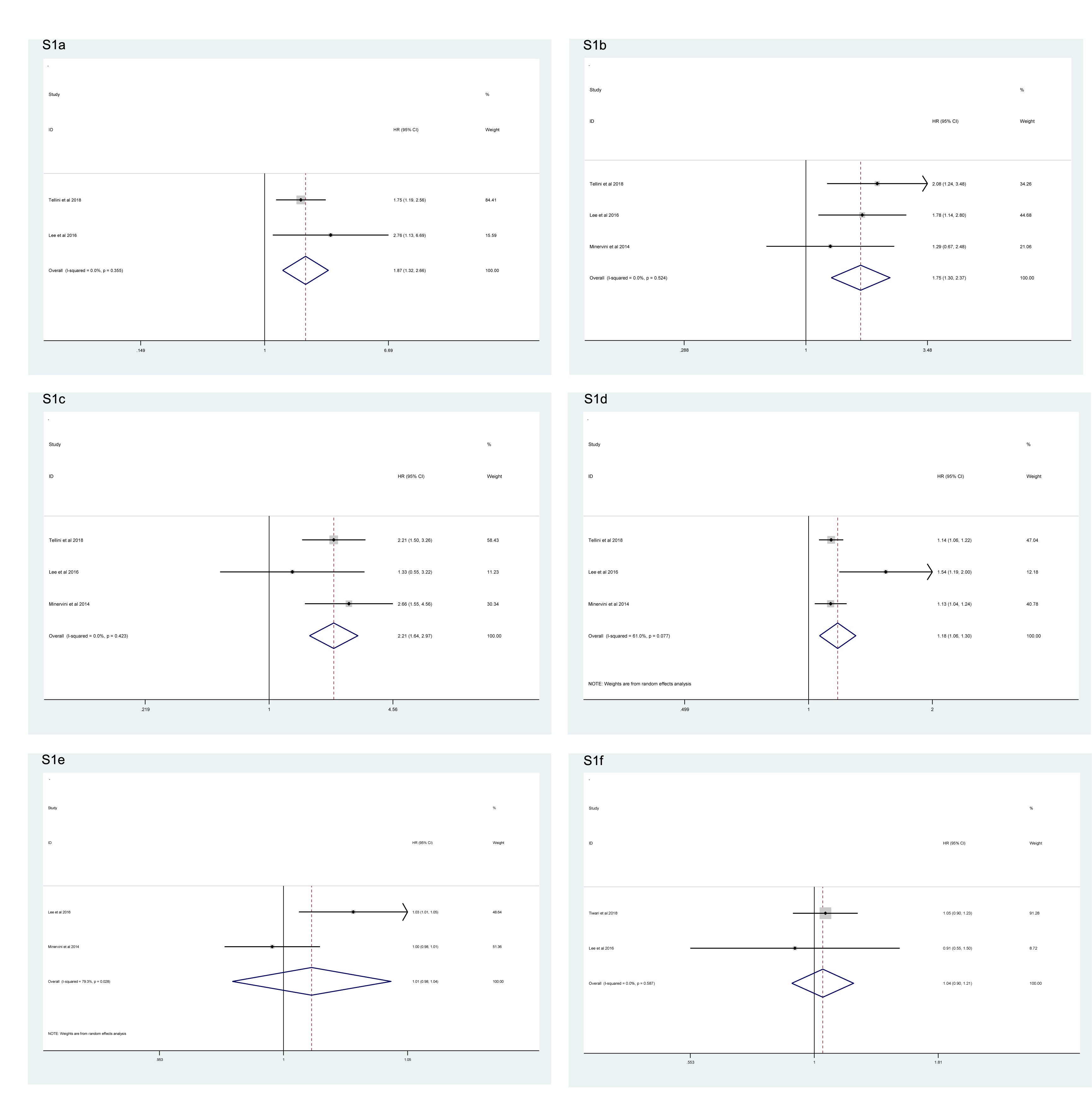

Supplement: Supplementary Figure S1 — Meta-analysis for the pooled HRs of RFS: (a) positive surgical margin; (b) higher Fuhrman grade; (c) higher pathological stage; (d) large tumor size; (e) age; (f) sex. [file Image_1.tif]

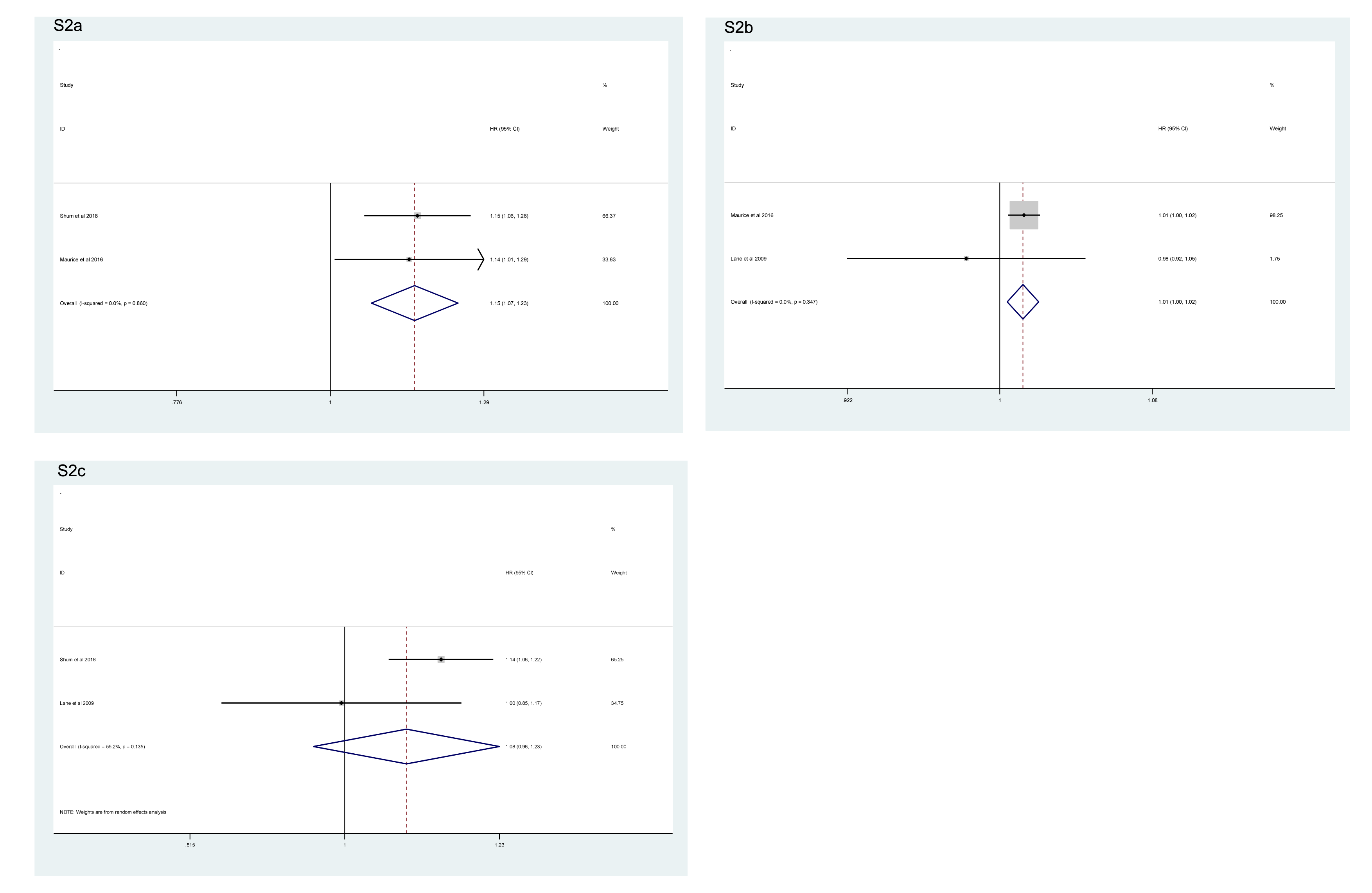

Supplement: Supplementary Figure S2 — Meta-analysis for the pooled HRs of OM: (a) positive surgical margin; (b) large tumor size; (c) sex. [file Image_2.tif]

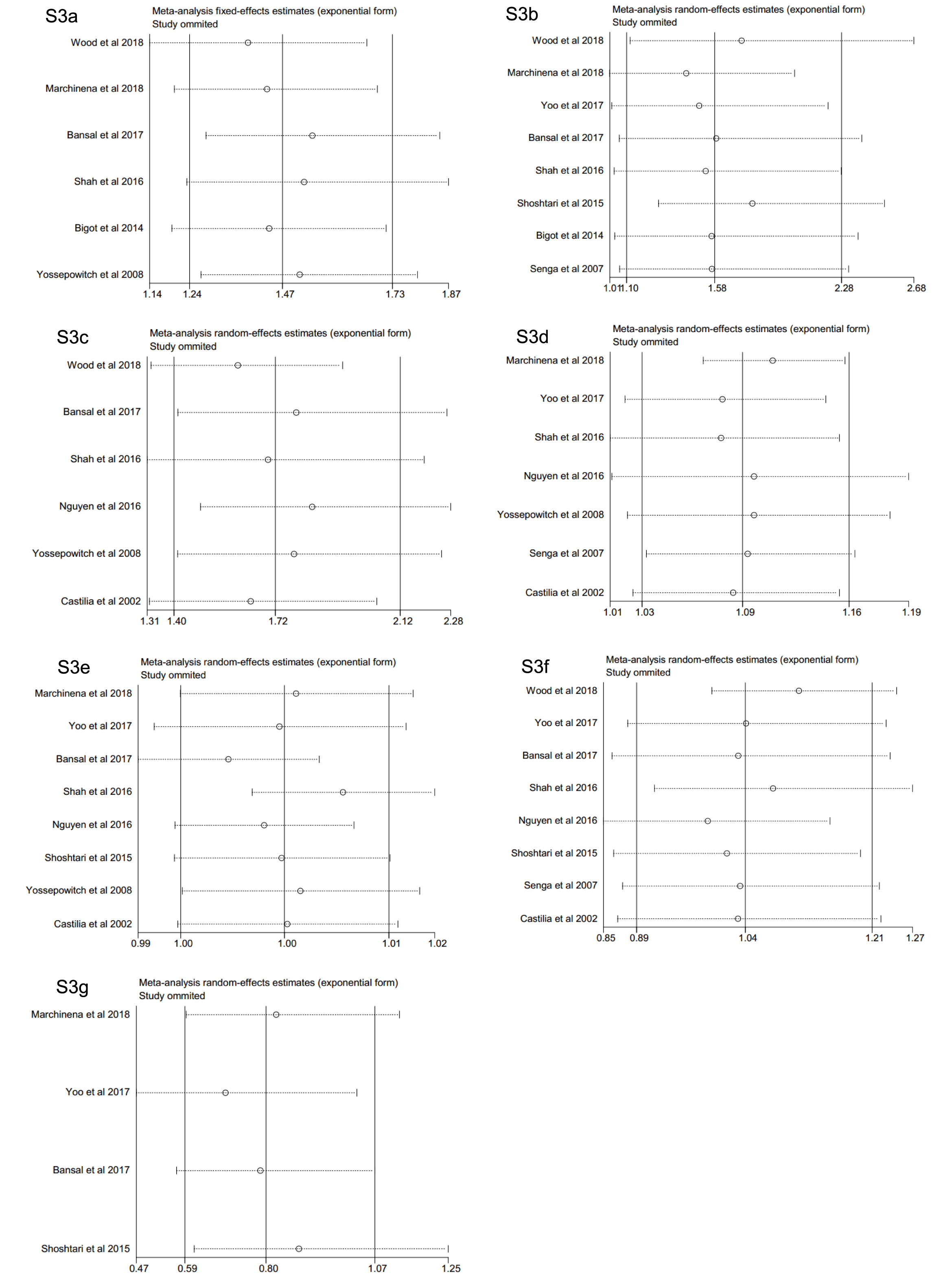

Supplement: Supplementary Figure S3 — Sensitivity analysis on the relationship between clinicopathological features and recurrence risk: (a) positive surgical margin; (b) higher Fuhrman grade; (c) higher pathological stage; (d) large tumor size; (e) age; (f) sex; (g) surgical approach. [file Image_3.tif]
