# Supplementary material for: The Clinicopathological Risk Factors in Renal Cell Cancer for the Oncological Outcomes Following Nephron-Sparing Surgery: A PRISMA Systematic Review and Meta-Analysis
Source: Front Oncol. 2020 Mar 6;10:286. doi: 10.3389/fonc.2020.00286 (PMC7067827; doi:10.3389/fonc.2020.00286)
Supplement: Supplementary file 4 [file Table_1.docx]

**Table S1. Quality assessment of cohort studies included in this meta- analysis**

| **Study** | **Representativeness of the exposed cohort** | **Selection of the unexposed cohort** | **Ascertainment of exposure** | **Outcome of interest not present at start of study** | **Control for important factor or additional factor** | **Outcome assessment** | **Follow-up long enough for outcomes to occur** | **Adequacy of follow-up of cohort** | **Total quality scores** |
| --- | --- | --- | --- | --- | --- | --- | --- | --- | --- |
| Wood et al. | ★ | ★ | — | ★ | ★ | ★ | ★ | ★ | 7 |
| Tellini et al | ★ | ★ | ★ | ★ | ★ | ★ | ★ | ★ | 8 |
| Shum et al | ★ | ★ | — | ★ | ★ | ★ | ★ | ★ | 7 |
| Marchinena et al | ★ | ★ | ★ | ★ | ★ | ★ | ★ | ★ | 8 |
| Yoo et al | ★ | ★ | — | ★ | ★ | ★ | ★ | ★ | 8 |
| Bansal et al | ★ | ★ | ★ | ★ | ★ | ★ | ★ | ★ | 8 |
| Shah et al | ★ | ★ | — | ★ | ★ | ★ | ★ | ★ | 7 |
| Nguyen et al | ★ | ★ | ★ | ★ | ★ | ★ | ★ | ★ | 8 |
| Maurice et al | ★ | ★ | — | ★ | ★ | ★ | ★ | ★ | 7 |
| Lee et al | ★ | ★ | ★ | ★ | ★ | ★ | ★ | ★ | 8 |
| Shoshtari et al | ★ | ★ | — | ★ | ★ | ★ | ★ | ★ | 7 |
| Minervini et al | ★ | ★ | ★ | ★ | ★ | ★ | ★ | ★ | 8 |
| Bigot et al | ★ | ★ | ★ | ★ | ★ | ★ | ★ | ★ | 8 |
| Lane et al | ★ | ★ | ★ | ★ | ★ | ★ | ★ | ★ | 8 |
| Senga et al | ★ | ★ | — | ★ | ★ | ★ | ★ | ★ | 7 |
| Castilia et al | ★ | ★ | — | ★ | — | ★ | ★ | ★ | 6 |
